# Searching for neurodegeneration in multiple sclerosis at clinical onset: Diagnostic value of biomarkers

**DOI:** 10.1371/journal.pone.0194828

**Published:** 2018-04-03

**Authors:** Lenka Novakova, Markus Axelsson, Clas Malmeström, Henrik Imberg, Olle Elias, Henrik Zetterberg, Olle Nerman, Jan Lycke

**Affiliations:** 1 Institute of Neuroscience and Physiology, Department of Clinical Neuroscience, Sahlgrenska Academy, University of Gothenburg, Gothenburg, Sweden; 2 Mathematical Sciences, Chalmers University of Technology and University of Gothenburg, Gothenburg, Sweden; 3 Institute of Neuroscience and Physiology, Department of Psychiatry and Neurochemistry, the Sahlgrenska Academy at the University of Gothenburg, Mölndal, Sweden; 4 Clinical Neurochemistry Laboratory, Sahlgrenska University Hospital, Mölndal, Sweden; 5 Department of Molecular Neuroscience, UCL Institute of Neurology, Queen Square, London, United Kingdom; 6 UK Dementia Research Institute at UCL, London, United Kingdom; Karolinska Institutet, SWEDEN

## Abstract

**Background:**

Neurodegeneration occurs during the early stages of multiple sclerosis. It is an essential, devastating part of the pathophysiology. Tools for measuring the degree of neurodegeneration could improve diagnostics and patient characterization.

**Objective:**

This study aimed to determine the diagnostic value of biomarkers of degeneration in patients with recent clinical onset of suspected multiple sclerosis, and to evaluate these biomarkers for characterizing disease course.

**Methods:**

This cross-sectional study included 271 patients with clinical features of suspected multiple sclerosis onset and was the baseline of a prospective study. After diagnostic investigations, the patients were classified into the following disease groups: patients with clinically isolated syndrome (n = 4) or early relapsing remitting multiple sclerosis (early RRMS; n = 93); patients with relapsing remitting multiple sclerosis with disease durations ≥2 years (established RRMS; n = 39); patients without multiple sclerosis, but showing symptoms (symptomatic controls; n = 89); and patients diagnosed with other diseases (n = 46). In addition, we included healthy controls (n = 51) and patients with progressive multiple sclerosis (n = 23). We analyzed six biomarkers of neurodegeneration: cerebrospinal fluid neurofilament light chain levels; cerebral spinal fluid glial fibrillary acidic protein; cerebral spinal fluid tau; retinal nerve fiber layer thickness; macula volume; and the brain parenchymal fraction.

**Results:**

Except for increased cerebral spinal fluid neurofilament light chain levels, median 670 ng/L (IQR 400–2110), we could not find signs of early degeneration in the early disease group with recent clinical onset. However, the intrathecal immunoglobin G production and cerebral spinal fluid neurofilament light chain levels showed diagnostic value. Moreover, elevated levels of cerebral spinal fluid glial fibrillary acidic protein, thin retinal nerve fiber layers, and low brain parenchymal fractions were associated with progressive disease, but not with the other phenotypes. Thin retinal nerve fiber layers and low brain parenchymal fractions, which indicated neurodegeneration, were associated with longer disease duration.

**Conclusions:**

In clinically suspected multiple sclerosis, intrathecal immunoglobin G production and neurofilament light chain levels had diagnostic value. Therefore, these biomarkers could be included in diagnostic work-ups for multiple sclerosis. We found that the thickness of the retinal nerve fiber layer and the brain parenchymal fraction were not different between individuals that were healthy, symptomatic, or newly diagnosed with multiple sclerosis. This finding suggested that neurodegeneration had not reached a significant magnitude in patients with a recent clinical onset of multiple sclerosis.

## Introduction

Multiple sclerosis (MS) is a chronic disease of the central nervous system (CNS) characterized by pathologic heterogeneity, with inflammatory and neurodegenerative features during all stages of the disease [1]. Although inflammation dominates the clinical course of early MS, this stage has also displayed early signs of neurodegeneration [2]. It remains unknown whether this early degeneration is an independent process in MS or whether it is secondary to inflammation. Understanding the mechanisms that cause neurodegeneration may be fundamental for developing therapies that halt this process, and thereby prevent progressive disability. The development of sensitive, accessible biomarkers of neurodegeneration could provide tools for exploring the pathophysiology of degeneration in MS. Furthermore, they may improve diagnostics, patient characterization, and predictions of disease severity in MS.

Among the known biomarkers of neurodegeneration, in this study, we investigated the cerebrospinal fluid (CSF) to determine the levels of neurofilament light chain (NFL), glial fibrillary acid protein (GFAP), and tau. We also assessed brain atrophy, the thickness of the retinal nerve fiber layer (RNFL), and the macula volume (MV). These biomarkers have the potential to reflect early neurodegeneration, which may facilitate predictions of the prognosis and the disease course. The RNFL and the total MV were associated with brain atrophy in MS [3–6], and peripapillary RNFL was one of the recommended measures for diagnosing and monitoring MS [7]. In a previous study, increased CSF NFL levels at diagnosis were associated with a worse prognosis [8], and CSF GFAP levels, brain atrophy, and thinning of the RNFL were correlated with disability and disease progression [3, 9–11]. These methods for assessing neurodegeneration are all widely accessible and are easily implemented in current MS care. Neurodegeneration is an important part of MS; thus, these methods were proposed to be included as part of no evidence of disease activity (NEDA) definition [12].

The purpose of this cross-sectional study was to examine the occurrence of early neurodegeneration and to determine the diagnostic value of degenerative biomarkers in individuals with a recent onset of suspected MS. This unselected cohort of individuals was referred to a regional Multiple Sclerosis Center over a period of 2 years. We will follow up this cohort to determine the predictive roles of the investigated degenerative biomarkers and their roles in monitoring disease activity, progression, and treatment response.

## Methods

### Patients and controls

At the Multiple Sclerosis Center of Sahlgrenska University Hospital, Gothenburg, Sweden, we consecutively included 271 patients with clinical features of suspected MS onset, between April 2014 and June 2016. The patients were referred to the Multiple Sclerosis Center from the hospital emergency department, primary care, and other specialist units and departments. After a diagnostic investigation, including an assessment of early neurodegeneration, the patients were classified into disease groups, as follows: patients with clinically isolated syndrome (CIS; n = 4) or early relapsing remitting multiple sclerosis with disease durations <2 years (early RRMS; n = 93); patients with RRMS with disease durations ≥2 years (established RRMS; n = 39); symptomatic controls without MS, but showing symptoms (SC; n = 89); and patients diagnosed with other diseases (OD; n = 46). Patients were diagnosed with MS when they fulfilled the revised McDonald criteria [13]. We retrospectively searched the local diagnostic registry in May 2017 and found 7 patients with MS that had been missed; thus, those patients were not included in the present survey. Consequently, the study population was considered an unselected incidence cohort of individuals with suspected MS that were referred to a Swedish Multiple Sclerosis Center. In addition, we included healthy controls (HC; n = 51) and patients with progressive multiple sclerosis (PrMS; n = 23) as negative and positive control groups, respectively. None of the patients or control subjects was treated with immunomodulatory or immunosuppressive treatment.

The OD group included patients with optic neuritis (ON; n = 11), myelitis (n = 9), radiologically isolated syndrome (n = 3), unspecified demyelinating disorders (n = 3), spinal disc herniation (n = 3), peroneal mononeuropathy (n = 2), polyneuropathy (n = 1), narcolepsy (n = 1), trigeminal neuralgia (n = 1), cerebral autosomal dominant arteriopathy (n = 1), non-arteritic anterior ischemic optic neuropathy (n = 1), hemifacial spasm (n = 1), neuromyelitis optica (n = 1), Bell´s palsy (n = 1), systemic lupus erythematosus with central nervous system involvement (n = 1), atypical facial pain (n = 1), neurogenic bladder disorder (n = 1), epilepsy (n = 1), neuroborreliosis (n = 1), and spinal arachnoid cyst (n = 1).

The SC group had normal neurological examinations and normal magnetic resonance imaging (MRI) of the brain. None of the HC group had any neurological signs or history of neurological disease. All patients and controls participated voluntarily in the study and provided written informed consent. The Regional Ethics Review Board in Gothenburg, Sweden, approved the study (Reference number 895–13).

### Diagnostic assessments

The clinical diagnostic work-up included a neurological examination performed by neurologists specialized in MS. A relapse was defined as an episode of neurological disturbance that lasted for at least 24 h, and that could not be better explained by another cause [13]. In CIS/early RRMS, 66 patients had relapse within 3 months prior to sampling, median 25 days (IQR 12–41). In established RRMS, 22 patients had relapse within 3 month prior to sampling, median 22 days (IQR 10–45). None of the patients was treated with corticosteroids prior to sampling. Disability was scored with the Expanded Disability Status Scale (EDSS) [14]. Disease duration was calculated from the first MS symptom onset. A standard MRI protocol for the brain used for diagnosing MS included intravenous gadolinium as a contrast agent to facilitate diagnostics. The examination was performed on a 3 Tesla MRI scanner, and it included T1, fluid attenuation inversion recovery (FLAIR), and T2 sequences, performed according to the Swedish guidelines [15]. The median time between sampling and MRI was 2 days (IQR 1–16).

### Assessment of early neurodegeneration

Samples of peripheral blood and CSF were obtained during the clinical assessments. These were analyzed to determine the cell count, albumin ratio, intrathecal immunoglobulin G (IgG) production, including IgG and/or the presence of oligoconal bands (OCB), and the levels of NFL, GFAP, and Tau. The samples were handled according to the consensus protocol of the BioMS-EU network for CSF biomarker research in MS [16]. All measurements were performed by certified laboratory technicians in the Clinical Neurochemistry Laboratory at the Sahlgrenska University Hospital. The concentration of NFL in CSF was measured with a sensitive sandwich ELISA method (NF-light® ELISA kit, UmanDiagnostics AB, Umeå, Sweden), according to the manufacturer’s instructions. The lower limit of quantification (LLoQ) of the assay was 31 ng/L. The intra- and interassay coefficients of variation were below 10%. The reference ranges of CSF-NLF at different ages were: <30 years, <380 ng/L; 30 to <40 years, <560 ng/L; 40 to <60 years, <890 ng/L; ≥60 years, <1850 ng/L. The concentration of GFAP in CSF was measured with an in-house ELISA, as previously described [17]. The LLoQ of the GFAP assay was 16 pg/mL. The respective intra- and interassay coefficients of variation were 4% and 8%, respectively. The reference ranges for CSF-GFAP at different ages were: 2 to <20 years, <175 ng/L; 20 to <60 years, <750 ng/L; ≥60 years, <1250 ng/L. The concentration of tau in CSF was measured with an ELISA (INNOTEST hTAU Ag, Fujirebio, Ghent, Belgium). The LLoQ of the assay was 75 ng/L, and intra- and interassay coefficients of variation were below 10%. The reference ranges for CSF-tau at different ages were: 18 to <45 years, <300 ng/L; ≥45 years, <400 ng/L. Synthetic MRI (SyMRI) software, version 8, was used to assess brain atrophy; this program automatically quantified the brain parenchymal fraction (BPF) and white and grey matter volumes. SyMRI is a novel, reproducible, rapid automatic method for calculating the BPF. Synthetic tissue mapping is based on absolute quantification of tissue relaxation rates (R1 and R2) and proton attenuation. It was strongly correlated with a manual segmentation technique, with a low coefficient of variation (0.45%) [18]. Optical coherence tomography (OCT) was performed by a trained nurse on a Topcon 3D OCT– 2000. OCT was used to measure the mean RNLF thickness and the mean MV. Patient eyes that had ON were excluded in the OCT analyses, because a history of ON can interfere with measurements of general neurodegeneration [19] we intended to study These patients were included when analyzing all other biomarkers.

### Statistical methods

Group comparisons were performed with the T-test for continuous variables. Correlations between biomarkers were estimated with the Spearman rank correlation. Correlations were visualized in correlation matrix plots, for all observations and for subgroups of individuals. The biomarkers were clustered and reordered with hierarchical clustering. We used “1 –the correlation” as a distance metric and the average link; i.e., we joined the particular pair of clusters with the smallest average distance between pairs of biomarkers. Thus, the biomarkers with smallest average distance had highest correlations and were depicted close to each other in the figures. The sign of the OCT biomarkers was changed prior to clustering; thus, higher values corresponded to greater neurodegeneration for all variables.

We used the principal component analysis to visualize high-dimensional data and identify patient clusters. Standardized data were decomposed into uncorrelated directions and sorted according to how well they explained the variance in the data. This illustrated distance and dependence between the studied groups.

Prediction analyses were performed to determine whether biomarkers could discriminate between SC, RRMS, and OD groups. All the biomarkers identified with the CSF, OCT, and SyMRI analyses were tested separately. For this purpose, we employed a classification tree model with gini impurity as splitting criterion. The size of the tree was selected based on a 10-fold cross validation to minimize the cross-validated misclassification error.

To remove the effect of age on the biomarkers in group comparisons and in prediction analyses, we performed age adjustments for variables that were significantly related to age (at 10% level) in the HC group. These variables included white and grey matter volumes, BPF, NFL, GFAP, tau, and the albumin ratio. Age adjustments were performed by subtracting the age trend observed with the HC data, which was estimated with linear regression, from the observed values in the variables of interest. Moreover, NFL, GFAP, tau, the albumin ratio, the number of lymphocytes, and the IgG index were log-transformed prior to analysis.

All statistical tests were performed at a 5% level of significance, without corrections for multiple testing. This justified by the exploratory nature of this study and the limited group sizes. Only complete cases were included in the cluster analysis, principal component analysis and prediction analysis.

Statistical analyses were performed with IBM SPSS Statistics 24 software (IBM Corp.) and R statistics software, version 3.4.2 [20]. Classification trees were fitted with the *rpart* package available in R, version 4.1–11 [21].

## Results

### Degenerative biomarkers in CSF

**Fig 1:**
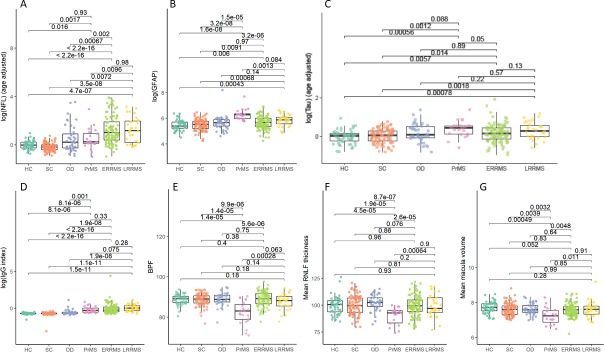
Comparison of biomarker levels among groups with and without multiple sclerosis. (A) NFL (age adjusted) levels, (B) GFAP levels, (C) tau levels, (D) IgG index, (E) BPF values, (F) RNFL thickness, and (G) macula volume in control groups (HC, SC) and in patients with OD, PrMS, CIS/early RRMS (with disease duration <2 years; ERRMS), and established RRMS (disease duration ≥ 2 years; LRRMS). Dots represent the log of the biomarker values or the tissue properties measured in each subject. Boxes are the IQRs and horizontal lines are the median values. Brackets indicate pairwise group comparisons with corresponding p-values. The age adjusted log(NFL) was calculated, as follows: log(NFL)– 4.24–0.040 * Age (y), as estimated by linear regression of the HC group. The age adjusted log(tau) was calculated, as follows: log(tau)– 5.75 + 0.015*Age (y), as estimated by linear regression of the HC group.

The characteristics of the patients and controls are presented in Table 1. All phenotypes of MS exhibited significantly elevated NFL (CIS/early RRMS, 63.2%; established RRMS, 63.9%; PrMS, 38.1%) compared to HC (4%) and SC (0%) (p<0.001). NFL levels were higher in the newly diagnosed RRMS group compared to the PrMS group, and they were within the normal range in the HC and SC groups (Fig 1A). The PrMS group had significantly higher GFAP levels compared to the HC, SC, CIS/early RRMS (all p<0.001), and established RRMS (p = 0.0013) groups. There was no significant difference in the levels of GFAP between the CIS/early RRMS and established RRMS groups. However, the newly diagnosed RRMS group had higher GFAP levels than the HC and SC groups (Fig 1B). Tau levels were lower in the HC and SC groups compared to the CIS/early RRMS group (p = 0.006 and p = 0.014, respectively), compared to the established RRMS group (p<0.001 and p = 0.002, respectively), and compared to the PrMS group (p<0.001 and p = 0.001, respectively). No significant differences in tau levels were found between patients with different MS phenotypes, although tau levels tended to be lower in the CIS/early RRMS group than in the PrMS group (p = 0.05; Fig 1C). The IgG index was higher in all phenotypes of MS compared to the HC, SC, and OD groups (Fig 1D).

**Table 1:** Characteristics of the patients and controls.

	CIS/RRMS<2yr (n = 97)	RRMS≥2yr (n = 39)	PrMS (n = 23)	SC (n = 89)	HC (n = 51)	OD (n = 46)
Age, yr, mean (range)	34 (18–62)	38 (19–64)	51 (36–67)	35 (17–56)	27 (20–49)	35 (17–58)
Gender, n, F/M	67/30	30//9	14//9	73/16	24/27	35//11
Disease duration, yr, mean (range)	0.2 (0–1.5)	8.4 (2–39)	14 (2–30)	0.3 (0–5)	0	0.3 (0–3)
EDSS, median (range)	2 (0–6)	2 (0–3.5)	6 (2.5–6)	1 (0–3.5)	0	2 (0–4.5)
Relapse within 3 months, n (%)	66 (68.0)	22 (56.4)	0 (0)	58 (65.2)	0 (0)	25 (50.0)
OCB or increased IgG index, n (%)	91 (93.8)	37 (94.9)	21 (91.3)	3 (3.4)	3 (5.9)	13 (28.3)

CIS = clinically isolated syndrome, RRMS = relapsing remitting multiple sclerosis, PrMS = progressive multiple sclerosis, SC = symptomatic controls, HC = healthy controls, OD = patients with other disease, F = female, M = male, EDSS = Expanded Disability Status Scale, OCB = oligoclonal bands, IgG = immoglobulin G

### Measures of brain volume with Synthetic MRI

The SyMRI data showed that the BPF was reduced in the PrMS group compared to the HC, SC, and newly diagnosed RRMS groups (p<0.001). There was no significant difference in BPF between the HC, SC, and newly diagnosed RRMS groups, although patients with CIS/early RRMS tended to have higher BPF values than those in the established RRMS group (p = 0.063; Fig 1E). White matter and grey matter volumes were not significantly different between the different MS phenotypes.

### Retinal nerve fiber layer and macula volume determined with optical coherence tomography

The PrMS group had thinner RNFLs and lower MVs compared to the HC, SC, and RRMS groups (p<0.001). No significant differences in the RNFL or MV were found between the HC, SC, and newly diagnosed RRMS groups (Fig 1F and 1G). None of the biomarkers for degeneration were increased in the SC or HC groups, and we did not find any signs of pathology in the SC group that could distinguish it from the HC group.

### Degenerative biomarkers associated with disability and disease progression

The disease duration and the EDSS were negatively correlated with the RNFL (r = -0.24, p = 0.0030 and r = -0.267, p<0.001, respectively) and the BPF (r = -0.358, p<0.001 and r = -0.366, p<0.001, respectively). The disease duration was positively correlated with the EDSS (r = 0.283, p<0.001) and the GFAP (r = 0.311, p<0.001).

### Exploring relationships between biomarkers

**Fig 2:**
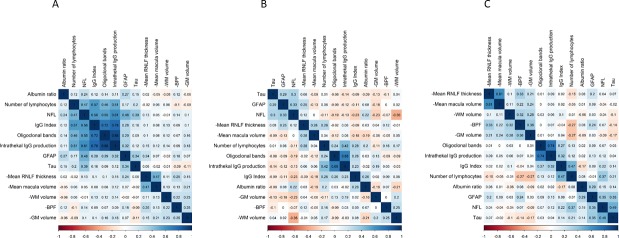
Correlation matrices for biomarkers. Matrices include (A) all patients and controls, (B) only patients with CIS/RRMS, and (C) only SC and HC groups. Blue: positive correlations; red: negative correlations. Spearman rank correlations are shown as numbers in the cells. The variables are clustered are reordered using hierarchical clustering.

Correlations between biomarkers are presented in Fig 2, for all patients and controls (Fig 2A), and separately for patients with CIS/RRMS (Fig 2B), and the HC group (Fig 2C). When all individuals in the analysis were included, two distinct clusters were observed. One cluster consisted of inflammatory biomarkers in the CSF (number of lymphocytes, IgG index, intrathecal IgG production, and OCB), the CSF NFL, and the CSF GFAP levels. The other cluster consisted of neurodegenerative biomarkers (brain volume measures, RNFL, and MV) (Fig 2A). When the CIS and RRMS groups were analyzed separately, a similar cluster pattern was observed (Fig 2B). However, the HC group showed a different cluster pattern. The cluster with inflammatory biomarkers in the CSF, the CSF NFL and the CSF GFAP, previously observed now appeared as two separate clusters. The cluster with brain volume measures, RNFL and MV was non-existent in HC (Fig 2C).

### Diagnostic value of biomarkers

**Fig 3:**
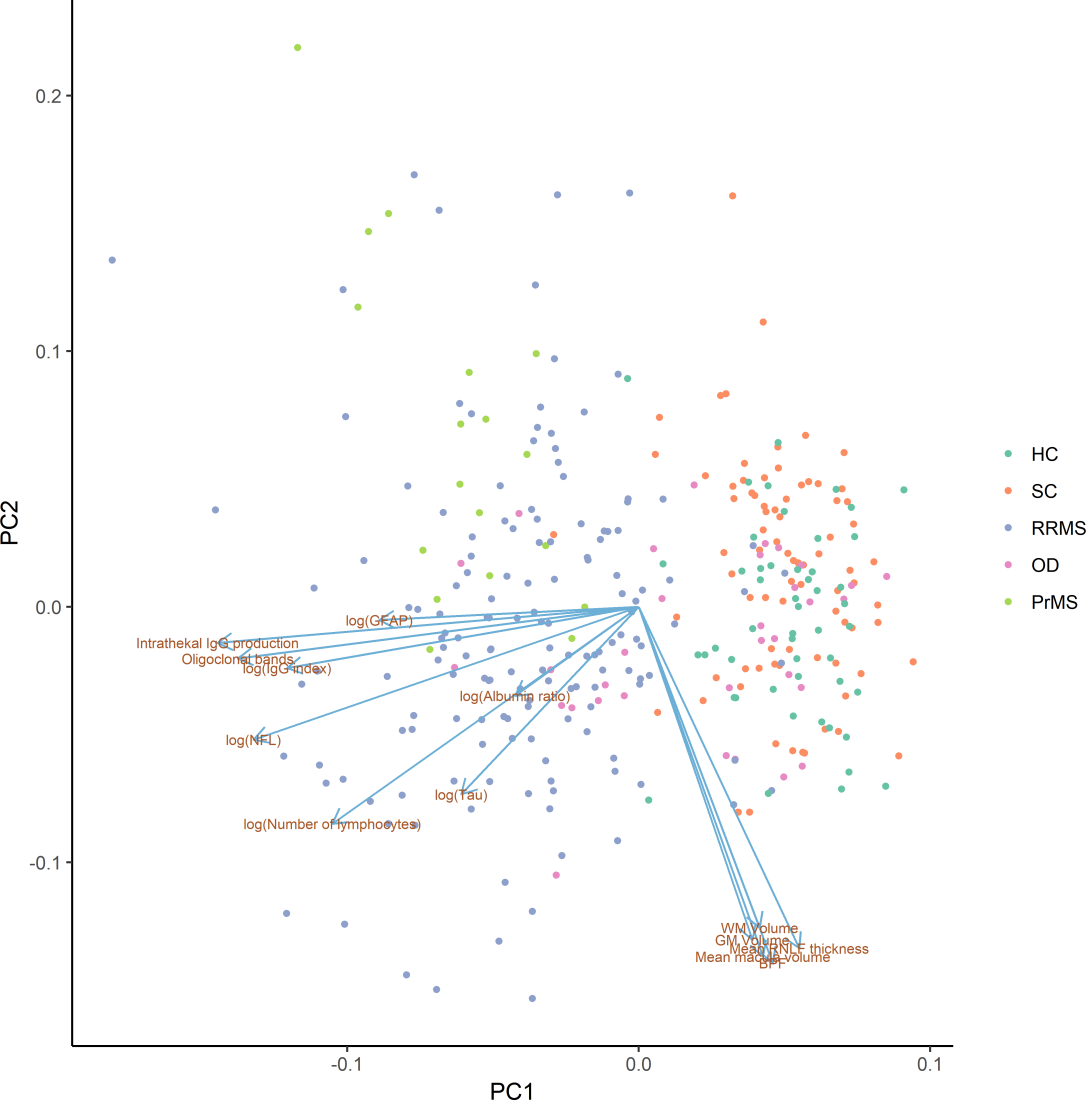
Scatterplot of data projected to the first two principal components (PC1 and PC2). PC1 and PC2 explain 27.7% and 15.7% of the variability in data, respectively (total 43.4%). The projections of the biomarkers onto the principal components are shown as arrows. The HC and SC groups are mainly separated from the RRMS and PrMS groups by the biomarkers measured in the CSF. The OD group overlaps with both of the other groups.

**Fig 4:**
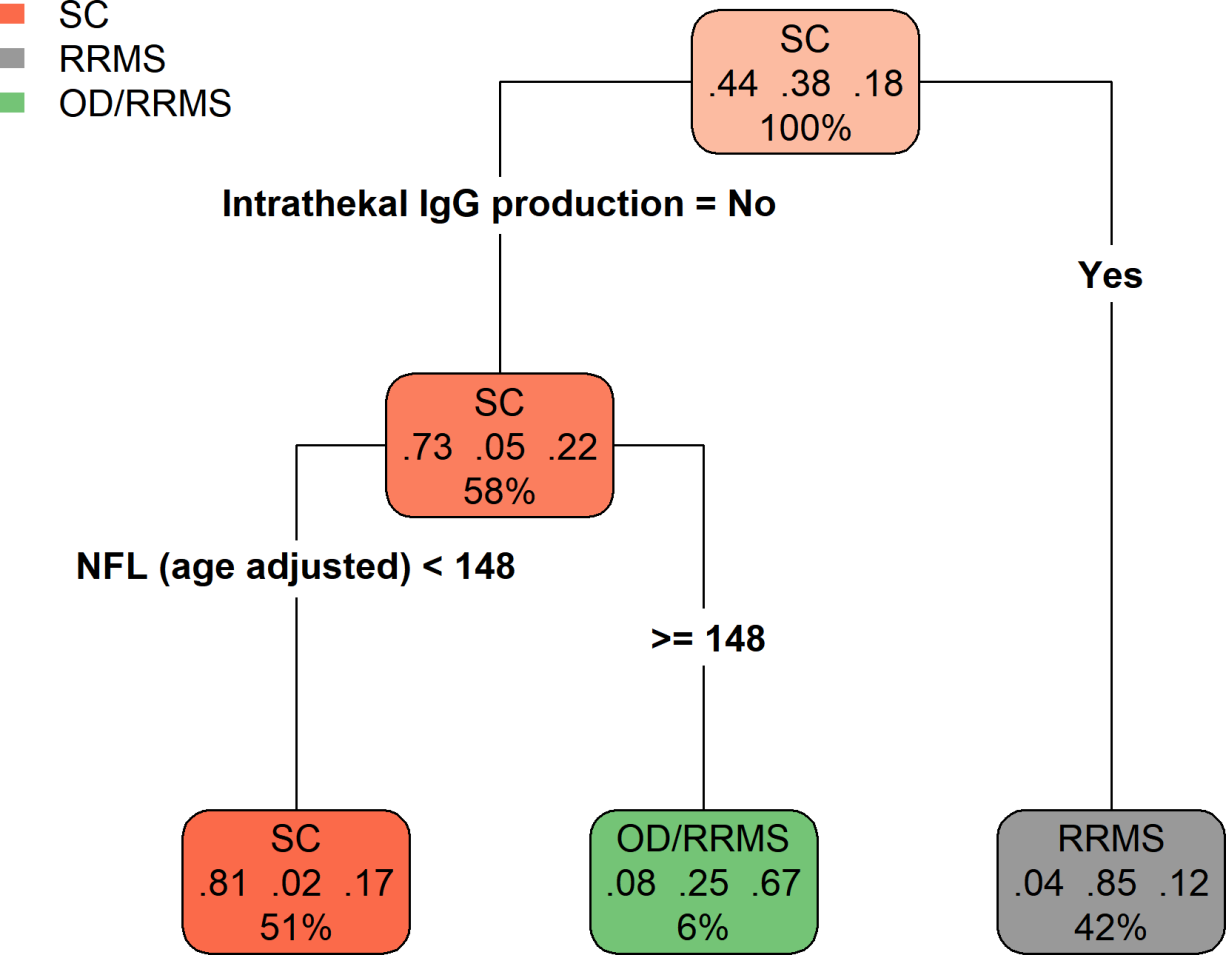
Classification tree model. Patients with previously undiagnosed RRMS (grey), the SC group (red), and the OD group (green) were classified by splitting the groups based on the detection of the indicated biomarkers. The class of the majority of patients in a group is shown at the top of each node. The proportions of individuals that belong to classes SC, RRMS, and OD are shown as fractions in the middle of each node. The percentage of the total number of individuals in each node is shown at the bottom of each node. This model makes two splits, the first is based on intrathecal IgG production, and the second is based on NFL levels (age-adjusted), with an optimal split in age adjusted NFL at 148 ng/L. Thus, individuals with no intrathecal IgG production and low levels of NFL were predicted to be SC (51%). Among the individuals that satisfied these two criteria, 81% were truly SC, 2% had RRMS, and 17% had some other disease. Only 6% of individuals exhibited no intrathecal IgG production and high levels of NFL. Of these, 8% were SC, 25% had RRMS, and 67% had OD. Finally, 42% of individuals exhibited only intrathecal IgG production. Of these, 4% were SC, 85% had RRMS, and 12% had OD. The calculation for age-adjusted NFL is: NFL– 203.3–15.9 * Age (y), as estimated by linear regression of the HC group.

The clusters of biomarkers observed with hierarchical clustering also appeared in principal component analysis, where CSF biomarkers mainly contributed to the first principal component and SyMRI biomarkers mainly to the second principal component. Furthermore, the CIS/RRMS and PrMS groups were clustered together and separated from the HC and SC groups along the first principal component, i.e, due to differences in the inflammatory biomarkers detected in the CSF (Fig 3). Next, we explored the diagnostic value of all biomarkers, compared to the known CSF biomarkers often used for diagnosing MS. In the unselected cohort of individuals with suspected multiple sclerosis, we measured the most important biomarkers for discriminating between SC, OD, and RRMS, based on the gain in accuracy when a split in the classification tree was performed. We found that the most important biomarkers were intrathecal IgG production, the IgG index, the presence of OCB, the NFL, and the number of lymphocytes. Using intrathecal IgG production only, 93% of the RRMS patients were identified (66 out of 71), with a false positive rate of 15% (3 SC and 9 OD). 96% of the SC (78 out of 81) were correctly predicted as such. Adding NFL, the sensitivity increased to 97% (69 out of 71 RRMS) and the false positive rate increased to 23% (4 SC and 17 OD) (Table 2). On top of intrathecal IgG production and NFL, no additional variables were selected in the final model. Similar error rates were observed with cross-validation as on the training data, indicating a stable fit. The final prediction model can be interpreted as a diagnostic RRMS scale with three scores corresponding to low (2%), mid (25%) and high (85%) probability of RRMS (Fig 4).

**Table 2:** Predicted and observed group membership using a classification tree model with intrathecal IgG production and NFL. The formula for age adjusted NFL is given by NFL– 203.3–15.9 * Age (y), as estimated by linear regression in healthy controls.

Decision rule	Observed		
	SC	RRMS	OD
SC: No intrathecal IgG production and Age adjusted NFL < 148	77	2	16
OD/RRMS:No intrathecal IgG production and Age adjusted NFL ≥ 148	1	3	8
RRMS/OD: Intrathecal IgG production	3	66	9

IgG = immoglobulin G, NFL = neurofilament light, RRMS = relapsing remitting multiple sclerosis, SC = symptomatic controls, OD = patients with other disease

The OCT and SyMRI biomarkers did not contribute to the discrimination between the patient groups, either when studied separately or when all biomarkers were included in the analysis.

## Discussion

In an unselected cohort of individuals with clinically suspected MS, we investigated whether early degeneration could be detected with methods that have become increasingly accessible in clinical practice. We found that, among all biomarkers of degeneration, only NFL seemed to have diagnostic value, and therefore, it could be considered in the diagnostic work-up for MS. We could not detect any other significant signs of degeneration in individuals with CIS or RRMS at clinical onset. Our results confirmed findings from previous studies, which showed that GFAP was increased in PrMS, but not associated with other disease courses of MS [9]. We also found that a low BPF, which indicates an increased rate of brain atrophy, was associated with long disease durations. However, the influence on BPF at clinical onset of CIS/MS was not sufficient to distinguish between HC, SC, and CIS/early RRMS; this finding suggested that neurodegeneration had not reached a significant magnitude in the early stages of the disease. Compared to those groups, patients with established RRMS and patients with PrMS had thinner RNFLs and lower MVs. Similarly, RNFL, and MV could not distinguish between HC, SC, and CIS/early RRMS groups. Thus, except for NFL, the biomarkers investigated in this exploratory study of early degeneration had low diagnostic value. Nevertheless, our results indicated that the other biomarkers may be useful for monitoring the rate of degeneration in MS.

We showed that CSF biomarkers of inflammation were correlated with each other. However, except for NFL and GFAP, the biomarkers of neurodegeneration were only weakly related to inflammation. These results supported the hypothesis of inflammatory-induced neurodegeneration, which postulated that inflammatory biomarkers are prominent and neurodegenerative biomarkers are essentially absent during the early stages of MS. In contrast, other neurodegenerative biomarkers were altered in more advanced stages of MS. Their usefulness for monitoring progression and therapeutic response has been demonstrated in several studies and trials previously [22–25].

Since the introduction of the McDonald diagnostic criteria of MS [26], the diagnostic value of CSF analyses has declined. However, in the recently published revision of these criteria, the detection of oligoclonal IgG bands selectively in the CSF has regained importance. This biomarker may be used as a substitute for clinical or MRI analyses for establishing dissemination in time [27]. In the present exploratory study, we confirmed the high value of intrathecal IgG production for the diagnosis of MS [28, 29]. We also showed that the NFL level could be used to improve diagnostics in patients negative for OCB. In previous studies, NFL could predict disease severity at disease onset [8, 30] and was correlated with the progression of brain atrophy [31]. Therefore, NFL levels may be useful in the diagnostic work-up as well as for prediction of the disease course of MS.

With imaging biomarkers, we did not find signs of early neurodegeneration in patients with recent clinical onset of CIS/RRMS. This was in contrast with previous studies, showing that brain atrophy is present during all stages of MS and disease courses including CIS/early RRMS [32, 33]. However, we could confirm previous findings that brain atrophy is correlated with disease duration and disability [23, 34]. The discrepancy between our results and those of other studies might be explained by differences in study populations and differences in methods for measuring brain volume. In previous studies, patients with CIS/early RRMS exhibited high numbers of lesions on MRIs [35, 36], while the corresponding patients of our study cohort had low number of MS lesions. Also, the use of automated segmentation methods for determining brain volumes differed among studies, and the results displayed varying degrees of similarity to the manual reference values [37]. In the present study, brain volume measures were determined with SyMRI, which was previously shown to be the most reliable method, compared to the manual reference [37]. The SyMRI method showed good accuracy in determining whole brain volumes in cross-sectional studies [18] and the lowest repeatability errors in brain volume, BPF, intracranial volume, and grey matter fraction [38]. Our results were consistent with those from another study, where no significant whole brain atrophy was found in patients with CIS at clinical presentation, compared to controls. Only specific brain areas showed significant atrophy, including the bilateral thalamic, hypothalamic, putamen, and caudate areas [39]. In line with the results obtained from SyMRI we could not show a significant atrophy of the peripapillary RNFL in patients with recent MS onset. We confirmed that the peripapillary RNFL was of similar thickness in patients with recent clinical onset of MS and HC [40].

The main strength of this study was the unbiased cohort, which comprised consecutive, unselected subjects with suspected MS referred to a Multiple Sclerosis Center. All patients with CIS/RRMS were untreated, but some patients with RRMS had a history of earlier disease events, which allowed comparisons between patients with different disease durations. All subjects were assessed with methods for quantifying neurodegeneration that are increasingly accessible and can be readily performed in the diagnostic work-up of MS. We explored how these methods could contribute to the diagnostics and characterization of disease course of MS. Moreover, our comprehensive matrices showed the relationships between the investigated biomarkers. The main limitation in this study was the cross-sectional design. Consequently, we could not evaluate predictive measures. However, the purpose of this study was to focus on the diagnostic work-up, with the aim of refining the diagnostics and stratification of patients with MS.

In conclusion, except for NFL, the studied biomarkers did not indicate degeneration in CIS and early RRMS and only the analyses of intrathecal IgG production and CSF NFL could improve the diagnostic work-up of MS. Newer methods, like SyMRI and OCT, did not add value to MS diagnostics, but they should be examined in prospective longitudinal studies to determine their usefulness for assessing degeneration in MS. Our results implied that the degree of neurodegeneration in CIS and early RRMS was limited, and that degeneration was apparently undetectable with several of current validated methods of CNS degeneration. Thus, our results support that inflammation precedes early degeneration and emphasizes the importance of early initiation of disease modifying therapies to halt the occurrence of excessive neurodegeneration in MS.

## Supporting information

S1 File BROMS171127 till statistikerna_1. (SAV)
